# Ties between reading faces, bodies, eyes, and autistic traits

**DOI:** 10.3389/fnins.2022.997263

**Published:** 2022-09-28

**Authors:** Marina A. Pavlova, Valentina Romagnano, Julian Kubon, Sara Isernia, Andreas J. Fallgatter, Alexander N. Sokolov

**Affiliations:** ^1^Department of Psychiatry and Psychotherapy, Tübingen Center for Mental Health (TüCMH), Medical School and University Hospital, Eberhard Karls University of Tübingen, Tübingen, Germany; ^2^IRCCS Fondazione Don Carlo Gnocchi ONLUS, Milan, Italy

**Keywords:** reading covered faces, point-light body motion, body language reading, face reading, gender, reading in the eyes, social cognition, autistic traits

## Abstract

While reading covered with masks faces during the COVID-19 pandemic, for efficient social interaction, we need to combine information from different sources such as the eyes (without faces hidden by masks) and bodies. This may be challenging for individuals with neuropsychiatric conditions, in particular, autism spectrum disorders. Here we examined whether reading of dynamic faces, bodies, and eyes are tied in a gender-specific way, and how these capabilities are related to autistic traits expression. Females and males accomplished a task with point-light faces along with a task with point-light body locomotion portraying different emotional expressions. They had to infer emotional content of displays. In addition, participants were administered the Reading the Mind in the Eyes Test, modified and Autism Spectrum Quotient questionnaire. The findings show that only in females, inferring emotions from dynamic bodies and faces are firmly linked, whereas in males, reading in the eyes is knotted with face reading. Strikingly, in neurotypical males only, accuracy of face, body, and eyes reading was negatively tied with autistic traits. The outcome points to gender-specific modes in social cognition: females rely upon merely dynamic cues while reading faces and bodies, whereas males most likely trust configural information. The findings are of value for examination of face and body language reading in neuropsychiatric conditions, in particular, autism, most of which are gender/sex-specific. This work suggests that if male individuals with autistic traits experience difficulties in reading covered with masks faces, these deficits may be unlikely compensated by reading (even dynamic) bodies and faces. By contrast, in females, reading covered faces as well as reading language of dynamic bodies and faces are not compulsorily connected to autistic traits preventing them from paying high costs for maladaptive social interaction.

## Introduction

Mandatory covering faces with medical masks may lead to difficulties in social perception and interaction (for comprehensive review, see Pavlova and Sokolov, 2022a). For achieving efficient social interaction during the COVID-19 pandemic, we are forced, therefore, to combine social signals from different sources such as the eyes (with a face hidden behind a mask) and bodies. This is particularly challenging for individuals with neuropsychiatric conditions such as autism spectrum disorders (ASD) characterized by aberrant social cognition already in the pre-pandemic period.

Face and body language reading is vital for efficient interpersonal exchanges. Examination of social competence by using dynamic input is of importance, since in daily-life social interaction and non-verbal communication we never deal with motionless static faces and bodies. Over the past half century, focus in research on social cognition (our ability to extract information about affects, drives, and intentions of our counterparts) has been shifted from traditional usage of static stimuli (primarily, photographs) to dynamic displays. Point-light movies of faces and bodies decrease the influence of other cues (such as gender, age, and other sources of structural information that may elicit certain perceptual biases) on our capacity for face and body reading.

Starting from the inspiring work of Canadian researcher John N. Bassili (1978, 1979), point-light dynamic faces (with a set of light dots placed on an invisible darkly-colored face) had been demonstrated to provide sufficient information not only for perceiving them as faces, but also for accurate facial affect recognition (e.g., Berry, 1990; Dittrich, 1991; Hill et al., 2003; Pollick et al., 2003; Atkinson et al., 2012; Bidet-Ildei et al., 2020; see also Dobs et al., 2018). Exaggeration of facial expressions relative to a neutral expression results in enhanced ratings of the emotion intensity, whereas changing the duration of an expression has a negligible effect on these ratings (Pollick et al., 2003). Distinct facial affect leads to different recognition levels, for example, angry facial expressions are recognized poorer than neutral or happy ones (Atkinson et al., 2012). Individuals with schizophrenia (SZ) can reliably recognize basic emotions (such as anger, fear, sadness, and happiness) from point-light faces, though they are less proficient than healthy controls (Tomlinson et al., 2006). Neurotypical perceivers can identify a speaker based on silent point-light facial information solely (Rosenblum et al., 1996; Jesse and Bartoli, 2018; Simmons et al., 2021), recognize emotions from visual-only point-light facial displays of singers (Quinto et al., 2014), and perform well not only on explicit but also on implicit facial affect recognition tasks (Bidet-Ildei et al., 2020). They also effectively use information in point-light displays when matching both unfamiliar and known faces (Bennetts et al., 2013). Already 7-month-old infants discriminate between angry and happy facial point-light expressions (Soken and Pick, 1992). Near-infrared spectroscopy (NIRS) shows that concentration of oxyhemoglobin (oxy-Hb) increases in the right temporal cortex of 5- to 8-month-old infants viewing point-light faces (Ichikawa et al., 2010; Ichikawa and Yamaguchi, 2012). Children aged 4 years recognize happy point-light faces, and 5–6-year-olds recognize a subtler facial expression of sadness (Doi et al., 2008). By 5 years of age, children reliably judge gender in point-light faces of persons engaged in interaction, though adults can also determine gender in faces reciting the alphabet (Berry, 1991).

Almost five decades ago, the point-light technique segregating perceptual signals available through body motion (BM) or *biological motion*, from other cues, had been introduced by the outstanding Swedish scholar from Uppsala University Gunnar Johansson (Johansson, 1973). A growing body of evidence shows that neurotypical individuals are rather competent in inferring emotions and dispositions of counterparts represented by point-light BM (e.g., Dittrich et al., 1996; Pollick et al., 2001; Atkinson et al., 2004; Heberlein et al., 2004; Clarke et al., 2005; Atkinson, 2009; Manera et al., 2010; Alaerts et al., 2011; Sokolov et al., 2011, 2020; Krüger et al., 2013; Actis-Grosso et al., 2015; Vaskinn et al., 2016). Effective body language reading is preserved in healthy aging, with particular tuning to displays portraying happiness (Spencer et al., 2016). Point-light gait can drive reliable judgments of personality traits such as approachability, neuroticism, trustworthiness, and warmth (Thoresen et al., 2012; see also Pavlova, 2012 on the Russian psychiatrist Pyotr B. Gannushkin who was reportedly able to recognize mental conditions of patients simply by observing their changing outline as they moved about in a dimly lit room).

Visual processing of BM and social cognitive abilities had been argued to be intimately tied (Pavlova, 2012). Indeed, individuals with neurodevelopmental and neuropsychiatric conditions (such as ASD, Williams-Beuren syndrome, and Down syndrome) and survivors of premature birth exhibiting aberrant processing of point-light BM also possess lower daily-life social competence (for reviews, see Pavlova, 2012; Pavlova and Krägeloh-Mann, 2013; Pavlova et al., 2021). Yet experimental data suggests that this association may be modulated by other factors such as gender (and age) as well as by methodological issues including task design and stimuli used. In earlier work of our group (Isernia et al., 2020), by using the same set of displays, i.e., identical visual input, task demands were directed either to body motion processing (determination of actors’ gender) or emotion recognition. In males only, BM processing was found to be tightly connected with body language reading. Yet, in 8–11-year-olds, inter-correlations between four tasks (determination of a point-light walker’s facing, detection of a point-light walker embedded into noise, labeling of actions of a stick moving figure, and person identification from moving style of a stick walking figure) are rather weak (Williamson et al., 2015), suggesting that diverse capabilities are engaged in performance.

Not only BM processing allies with body language reading, but effective body language reading buddies with other social skills. For example, both revealing identity of point-light dancers and estimations of emotional expression intensity correlate with self-reported empathy (Sevdalis and Keller, 2011, 2012). Confidence in emotion perception in point-light displays varies with the ability to perceive one’s own emotions (Lorey et al., 2012). Body language reading ties not only with the more basic ability for discrimination between point-light canonical and scrambled BM displays, but also with accuracy on the Reading the Mind in the Eyes Test, RMET (Alaerts et al., 2011). In a sample of neurotypical adults predominated by females, efficiency of BM processing (such as facing detection of a point-light walker) is associated not only with performance on the RMET, but also with Autism Quotient (AQ), Empathy Quotient (EQ), and Cambridge Face Memory Test scores (Miller and Saygin, 2013). Even in children aged 7–12 years, BM detection is correlated with both reading in the eyes (as assessed by the RMET) and inferring of mental states based on understanding of stories (Strange Stories test; White et al., 2009), but performance on the RMET and Strange Stories test is not connected to each other (Rice et al., 2016). Therefore, in accord with earlier expectations (Pavlova, 2012), BM processing may be considered a basis for linking varied facets of social cognition. Curiously, even characteristics of social networks (such as a social network size defined as a number of peers heavily involved in daily communication) are reported to correlate with functional magnetic resonance imaging (fMRI) brain activation in response to point-light BM over key areas of the social brain such as the STS, superior temporal sulcus (Dziura and Thompson, 2014; Kirby et al., 2018).

Gender (a social construct) and sex (a neurobiological one) of observers are essential for performance on a wide range of social cognition tasks tapping bodies, faces, and eyes reading (Pavlova et al., 2010; Kret et al., 2011; Sokolov et al., 2011; Kirkland et al., 2013; Krüger et al., 2013; Pavlova et al., 2015, 2016, 2020; He et al., 2018, Dodell-Feder et al., 2020; Isernia et al., 2020; Kynast et al., 2021; see Pavlova and Sokolov, 2022b, for a most recent analysis of reading in the eyes). In the same vein, female but not male common marmosets (*Callithrix jacchus*) are reported to exhibit curiosity to point-light biological motion (Brown et al., 2010). Recently, gender/sex of observer is reported to affect reading covered faces, in particular, subtle emotional expressions (Carbon, 2020; Calbi et al., 2021; Grundmann et al., 2021; Proverbio and Cerri, 2022; for review, see Pavlova and Sokolov, 2022a). Magnetoencephalography (MEG) and fMRI reveal profound sex differences in the neural circuits underpinning point-light BM processing (Anderson et al., 2013; Pavlova et al., 2015; Jack et al., 2021). Females exhibit higher accuracy in recognition of point-light actions (such as jumping on the spot), and they are faster in discrimination of emotional from neutral locomotion (Alaerts et al., 2011). Yet gender differences in reading of body language (emotional locomotion and knocking on the door) are modulated by the portrayed emotion and actor gender (Sokolov et al., 2011; Krüger et al., 2013). Moreover, women surpass men in the recognition of neutral knocking (Sokolov et al., 2011). In females, but not males, body language reading is associated with mindreading in the eyes (Isernia et al., 2020). As pointed out earlier (Pavlova, 2012, 2017a,b; Duchesne et al., 2020), gender/sex impact can be of substantial value not only for a better conceptualization of social cognition, but also for understanding neuropsychiatric conditions most of which are gender/sex-specific.

**FIGURE 1:**
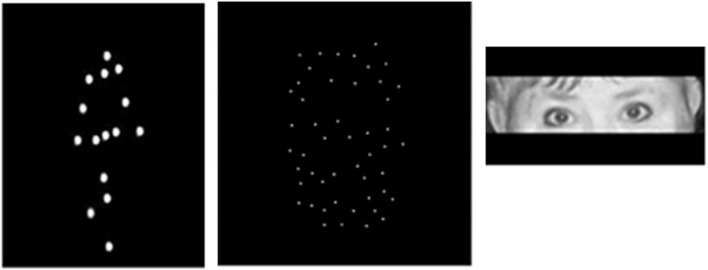
Illustration of stimuli used. From left to right: a static frame from dynamic sequence exemplifying locomotion as a set of dots placed on the main joints and a head of an invisible actor (a walking person is seen facing right in intermediate position between the frontal and sagittal views); a frame from dynamic sequence representing a point-light face of a female actor expressing anger; illustration of stimuli used for studying reading in the eyes [From Pavlova and Sokolov (2022a) with permission of Oxford University Press, and permission and written agreement of the poser].

Covering faces with masks leaves a comparable amount of visual information for face reading as the RMET (a set of photographs of a pair of eyes along with the surrounding part of a face including hairstyle; Pavlova and Sokolov, 2022a,b; Figure 1) does. Most recent experimental work indicates that RMET performance predicts accuracy of facial affect recognition of masked faces (Swain et al., 2022). Clarifying the issue of how masks affect face reading in real life, where we deal with dynamic faces and have *entrée* to additional social signals such as body language, warrants rigorous experimental work (Pavlova and Sokolov, 2022a). In real life, we usually cope with plentiful and often redundant social information that helps to prevent paying high costs for maladaptive or misleading social interaction. It was shown, for example, that the influence of face masks on recognition of emotions (anger, happiness, sadness, and fear) is diminished (or even negligible) when static whole body is present (Ross and George, 2022). Moreover, as the lack of information from masked faces may be compensated by other sources such as dynamic bodies, it is worthwhile to study whether, and, if so, how the abilities for face, body, and eyes reading are connected to each other.

In the present work, we examined: (i) whether the abilities for reading of dynamic faces and bodies are intimately tied; (ii) whether, and, if so, how, this link is gender-specific; and (iii) whether face reading and body reading are related to other social skills such as reading language of the eyes. Based on the outcome of earlier work (e.g., Miller and Saygin, 2013; Gökçen et al., 2014; Baltazar et al., 2021), we expected that efficiency of body, face, and eyes reading will be related to autistic traits expression. Neurotypical females and males accomplished a body language reading task along with a face reading task. They had to infer emotional content of displays. Furthermore, participants were administered the Reading the Mind in the Eyes Test, Modified (RMET-M) and Autism Quotient (AQ) questionnaire.

## Materials and methods

### Participants

Fifty participants (26 females and 24 males; aged 19–31 years) were involved in the study. The data set of one male participant was excluded from further data processing, since he turned out to have a history of psychiatric conditions. This left the data of 49 (23 males) participants. None of them had head injuries, a history of neuropsychiatric disorders (including ASD, SZ, and depression), or regular drug intake (medication). Males were aged 26.13 ± 2.96 years (mean ± standard deviation, SD), and females 24.96 ± 3.5 years (*t*(47) = 0.81, *p* = 0.212, two-tailed, n.s.). As performance on the RMET-M (German version, modified; for details, see below) requires language command of high proficiency, German as native language was used as one of the inclusion criteria. All observers had normal or corrected-to-normal vision. Participants were tested individually, and were naïve as to the purpose of the study. None had previous experience with such displays and tasks. The study was conducted in line with the Declaration of Helsinki and approved by the local Ethics Committee at the University of Tübingen Medical School. Informed written consent was obtained from all participants. Participation was voluntary, and the data sets were processed anonymously.

### Face reading: Point-light faces

For this task (inferring of emotions from face motion, face-motion-emotion, FME), participants were presented with a set of point-light black-and-white animations portraying face motion of female and male protagonists expressing happiness and angriness. Display production is described in detail elsewhere (Atkinson et al., 2012). The stimuli were kindly shared with us by Dr. Anthony Atkinson. In brief, 50 small white dots were positioned in a quasi-random order on an actor face. To ensure an even distribution of the dots, the face was divided into four quadrants, with the tip of the nose as a center, where two imaginary lines, horizontal and vertical, met. Each quadrant contained approximately the same number of white dots. The quasi-random placement minimized availability of structural information, such as from areas of the lips, cheeks or eyebrows. No dots were placed on the eyelids. Still, some static form cues could not be prevented such as dark regions at the position of eyes and a mouth’s opening. The displays had been proven for recognizability in behavioral and neuroimaging studies (e.g., Atkinson et al., 2012).

The videos of 6 (3 female/3 male) actors with happy and angry expressions were presented in 3 separate runs with a short break between them. In total, each experimental session consisted of a set of 108 trials (6 actors [3 female/3 male] × 2 emotions [happy/angry] × 3 displays for each emotion by each actor × 3 repetitions of each stimulus). In a two-alternative forced choice (2AFC) paradigm, participants had to indicate (by pressing one of two respective keys) facial affect (happy or angry). Each video lasted 2 s. Participants were asked to respond right after stimulus offset. During an inter-stimulus interval (ISI; after stimulus offset till onset of the next stimulus right after participant’s response) that was randomly jittered between 3 and 5 s, a white fixation cross was displayed in the center of the screen. If participants failed to respond within this period, the next trial started automatically.

### Body language reading: Point-light locomotion

For inferring of emotion from point-light BM, body-motion-emotion (BME) task, participants were presented with a set of point-light black-and-white animations portraying human locomotion. Display production is described in detail elsewhere (Ma et al., 2006; Krüger et al., 2013). The displays were built up by using the Motion Capture Library (N Stage, Pinewood Studios, Iver Heath, Buckinghamshire, United Kingdom). In brief, recording was performed using a 3D position measurement system at a rate of 60 Hz (Optotrak, Northern Digital Inc., Waterloo, ON, Canada). The matrix data for each frame was processed with MATLAB (The Mathworks Inc., Natick, MA, United States) into a video sequence. Each display consisted of 15 white dots visible against a black background (Figure 1). The dots were placed on the shoulder, elbow, and wrist of each arm; on the hip, knee, and ankle of each leg; and on the head, neck, and pelvis of a body. As we intended to make tasks demanding and expected more pronounced effects with brief stimulus duration, each movie lasted for 2 s that corresponded to one walking cycle consisting of two steps. During locomotion, a walker was seen facing right in the intermediate position of 45° between the frontal and sagittal views. As the sagittal view is often considered neutral in respect to possible social interactions, and the frontal view is reported to elicit ambiguous (facing either backward or toward an observer) and often gender-dependent impressions of locomotion direction (Pollick et al., 2005; Brooks et al., 2009; Schouten et al., 2010, 2011), the intermediate trajectory of locomotion was used. For creation of left-facing stimuli, we rotated the videos to 90° horizontally. The walking figure was pelvis-fixed to the middle of the screen. Female and male actors walked either with angry or neutral expression. For avoiding variability in emotion portrayal, several sets of neutral and angry stimuli were produced from the same actors.

The stimuli were selected from a previous study of our group (Isernia et al., 2020): we excluded movies of one female and one male actor that were the least recognizable ones. As a result, the videos of 4 (2 female/2 male) actors facing either right or left were presented in 3 separate runs with a short break between them. In total, each experimental session consisted of a set of 144 trials (4 actors [2 female/2 male] × 2 emotions [neutral/angry] × 2 facing directions [left/right] × 9 [3 repetitions of each stimulus × 3 runs]). Participants were asked to respond upon each stimulus offset. In a 2AFC paradigm, participants had to indicate by pressing one of two respective keys the emotional content of locomotion (angry/neutral). During an ISI (after stimulus offset till onset of the next stimulus right after the participant’s response) that randomly varied between 3 and 5 s, a white fixation cross was displayed in the center of the screen. If the participant failed to respond within this period, the next trial started automatically.

### Reading the Mind in the Eyes Test, modified

A computer version of the RMET-M (M, modified) was additionally administered to all participants. This test is described in detail elsewhere [Baron-Cohen et al., 2001a; see also most recent analysis by Pavlova and Sokolov (2022b)]. In brief, the original standard version of the RMET consists of 36 black-and-white photographs of female and male eyes along with a corresponding face part expressing a certain emotional or affective state. On each trial, participants had to choose among four alternative descriptions (adjectives simultaneously presented on the screen) including the correct one that corresponded with the image. We modified the RMET German version in such a way that, first of all, it did not as heavily rely on language capabilities as the standard one. To this end, instead of four adjectives we used only two of them (one correct and one incorrect). For example, for the item with four response options [besorgt/alarmed (correct) – ernst/serious – beschämt/ashamed – verblüfft/bewildered (all three incorrect)], we chose [besorgt (correct) – ernst (incorrect)]. This also led to shortening decision making time and, respectively, response time, which is of importance for MEG recording with patients at a later time point. Second, we selected 16 photographs out of original 36 to make the set of stimuli balanced in respect to the number of (i) female and male photographs (8 female/8 male), and (ii) positive and negative affective expressions (8 positive/8 negative). In addition, on the basis of our previous research with the standard RMET version (Isernia et al., 2020), we selected the photographs on which reading in the eyes was most difficult in order to retain individual variability. Each experimental session consisted of 80 trials (16 photographs × 5 repetitions) presented in a pseudorandomized order. Each image was exposed for 2 s. Then two words (correct and incorrect responses) appeared on the right and left sides of a black screen. Participants were asked to respond as accurately but also as fast as possible upon stimulus offset (with a time limit of 12 s). After each response, during an ISI that randomly varied between 2 and 3 s, a white fixation cross was displayed in the center of the screen. If participants failed to respond, the next trial started automatically. The whole experimental session (consisting of all three tasks: body reading, face reading, and the RMET-M) took about 40–45 min per participant. For all three tasks, no immediate feedback was given regarding performance.

### Autism quotient questionnaire

The AQ questionnaire for ages 16 and up, developed by Simon Baron-Cohen and colleagues (Baron-Cohen et al., 2001b), is intended to assess the expression of autistic traits by self-estimation. The questionnaire comprises 50 items, or statements, such as “*I prefer to do things with others rather than on my own*.” For each statement, participants have to indicate how strongly that statement applies to her or him using four response options “*Definitely agree – Agree – Disagree – Definitely disagree*.” The maximal score of autistic traits expression is 50. Yet the response to each statement (item) is then scored in a binary fashion (either 0 or 1). The items with positive (agreement) or negative (disagreement) responses are balanced in the AQ questionnaire. The statements intend to cover five domains characterizing autistic traits expression, including social competence, attention shifting, and focus on detail. In the present study, the AQ questionnaire version psychometrically evaluated and adapted to the German population had been used (Freitag et al., 2007). Some statements of the AQ do not take into account changes in preferences elicited by aging or educational status rather than by personality traits. For instance, for the statement “*I would rather go to the library than to a party*,” older people as well as persons with higher educational status are generally more likely to provide a positive response than the youth and people with lower educational status. The present study comprises a rather homogenous group of students of comparable age and education, and, therefore, these factors are unlikely to affect their response choices.

### Data analysis

Inferential data processing was performed by using JMP software (version 13; SAS Institute; Cary, North Carolina, United States.). All data sets were first routinely assessed for normality of distribution by Shapiro-Wilk tests with subsequent uses of either parametric (such as analysis of variance, ANOVA, Student *t*-test, Pearson product moment correlation) for normally distributed data or, otherwise, non-parametric (such as Mann–Whitney *U*-test, Spearman rank correlation) statistics. For not normally distributed data sets, additionally to means and SDs, medians (Mdns) and 95% confidence intervals (CIs) are reported throughout the text.

## Results

### Face and body language reading

Individual rates of correct responses on both dynamic point-light tasks (inferring emotions either from face motion, FME, or from body motion, BME) were submitted to a mixed model 2 × 2 repeated-measures ANOVA with a within-subject factor Task (FME/BME) and a between-subject factor Observer Gender (female/male). The outcome revealed a main effect of Task (*F*(1;48) = 423.64, *p* < 0.001, effect size, *eta squared* η^2^ = 0.815; with greater accuracy on revealing emotions from dynamic faces than bodies) and a significant Task × Observer Gender interaction (*F*(1;48) = 40.65, *p* = 0.047, effect size, η^2^ = 0.297; with greater accuracy of females on the BME task and no gender difference on the FME task). The main effect of Observer Gender was non-significant (*F*(1;48) = 12.82, *p* = 0.26, n.s.). *Post hoc* analysis (using Tukey honestly significant difference, HSD, tests) indicated a lack of gender differences in accuracy of face reading (FME task: 0.74 ± 0.09 for females, 0.76 ± 0.07 for males; *t*(47) = 0.71, *p* = 0.479, n.s., all tests corrected for multiplicity), but an advantage of females on body reading (BME task: 0.67 ± 0.07 for females, 0.62 ± 0.09 for males; *t*(47) = 2.54, *p* = 0.014; effect size, Cohen’s *d* = 0.625). Females were also faster on correct responses in both tasks [FME: for females, 0.670 ± 0.261 s (Mdn, 0.574 s, 95% CI, from 0.570 to 0.770 s); and for males, 0.812 ± 0.259 s; Mann–Whitney test, *U* = 195, *p* = 0.039, two-tailed, effect size, *d* = 0.624; BME: for females, 0.580 ± 0.196 s, and for males, 0.759 ± 0.196 s; *t*(47) = 3.23, *p* = 0.002, two-tailed; effect size, *d* = 0.913].

**FIGURE 2:**
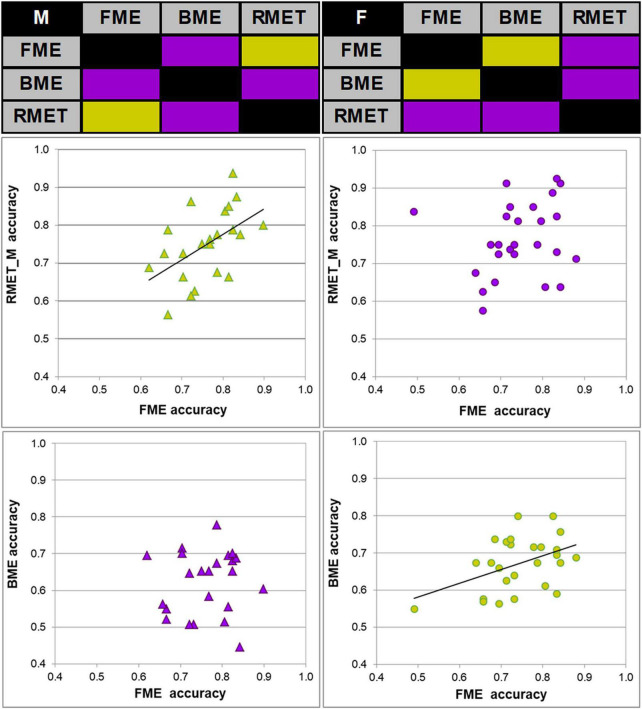
Links between accuracy of face and body reading through point-light biological motion, and performance on the Reading the Mind in the Eyes Test, modified (RMET-M), for female and male participants. Correlation matrices between accuracy of performance (correct response rate) on inferring emotions from faces (FME), bodies (BME), and the RMET-M for males (top left) and females (top right). Significant correlations (Pearson product moment correlations, two-tailed; *p* < 0.05) are color-coded by green, non-significant correlations by violet. Correlations between the FME and RMET accuracy in males (left middle panel, green diamonds) and between the FME and BME accuracy in females (right bottom panel, green circles) were significant.

**FIGURE 3:**
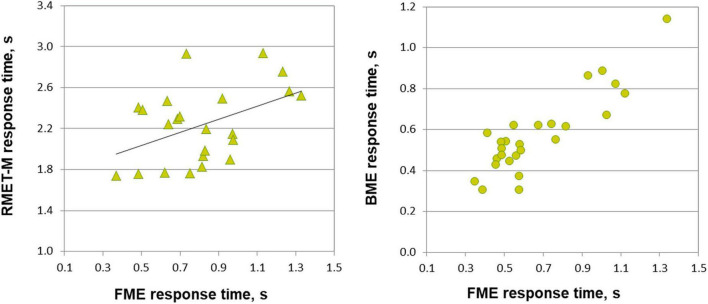
Relationship between response time on emotion through face motion (FME), body motion (BME) tasks, and RMET-M for female and male participants. In males (left panel, triangles), response time for correct responses on the FME task correlates with response time on the RMET-M (Pearson correlation). In females (right panel, circles) response times of correct responses on the FME and BME tasks correlate with each other (Spearman correlation, *p* < 0.001).

Most important for the purpose of the present work, face and body language reading were related to each other in a gender-specific manner. In females, accuracy of inferring emotions through point-light face (FME task) and body (BME task) were positively linked (Pearson product moment correlation, *r*(24) = 0.438, *p* = 0.025; effect size, Cohen’s *q* = 0.47), whereas no such association occurred in males (*r*(21) = 0.025, *p* = 0.91, n.s.; Figure 2). In females, also processing speed (correct response time) of face and body language reading were strongly allied with each other (Spearman’s rho, ρ(24) = 0.759, *p* < 0.001, effect size, *q* = 0.994), though this link occurred also in males (*r*(21) = 0.830, *p* < 0.001, effect size, *q* = 1.188; Figure 3).

### Link of face and body reading with reading in the eyes

The RMET-M was administered for addressing the issue of whether face reading and body language reading are connected to other social cognitive abilities such as reading in the static eyes. Based on earlier reports with the standard RMET (e.g., Kirkland et al., 2013; Baron-Cohen et al., 2015; Dodell-Feder et al., 2020; Kynast et al., 2021; for recent review, see Pavlova and Sokolov, 2022b) including the recent work of our own group (Isernia et al., 2020), we anticipated females to be more proficient on the RMET-M. Contrary to our expectations, however, accuracy of females and males was comparable (0.77 ± 0.10 for females, and 0.75 ± 0.09 for males; *t*(47) = 0.19, *p* = 0.288, one-tailed). Yet females surpassed males in processing speed, responding much faster (for females, 1.969 ± 0.390 s (Mdn, 1.949 s, 95% CI, from 1.819 to 2.119 s), for males, 2.236 ± 0.368 s; *U* = 185, *p* = 0.023, two-tailed; effect size, *d* = 0.69). Most startling within the framework of the present study is the outcome indicating that reading in the static eyes is gender-specifically related to reading dynamic faces. In males, accuracy on both tasks correlated with each other (*r*(21) = 0.506, *p* = 0.014; effect size, *q* = 0.557), whereas such bond was absent in females (*r*(24) = 0.195, *p* = 0.340, n.s.; Figure 2). Similarly, processing speed (as measured by correct response time) correlated between the RMET-M and FME tasks in males (*r*(21) = 0.450, *p* = 0.03; effect size, *q* = 0.485; Figure 3), but not in females (ρ(24) = 0.341, n.s.).

Based on earlier work (Alaerts et al., 2011; Miller and Saygin, 2013; Isernia et al., 2020), we expected to find a positive tie between accuracy of body language reading and mindreading in the eyes as measured by the RMET-M, at least, in female participants. Yet in both females and males, correlations between recognition accuracy on these tasks turned out to be non-significant (for females, *r*(24) = 0.235, *p* = 0.248; for males, *r*(21) = 0.207, *p* = 0.343, n.s.). Yet correct response time on the RMET-M and BME tasks correlated with each other both in females (ρ(24) = 0.420, *p* = 0.003; effect size, *q* = 0.448) and in males (*r*(21) = 0.607, *p* = 0.002; effect size, *q* = 0.704).

### Link of face, body, and eyes reading with autistic traits

In our sample of neurotypical individuals, the AQ scores were in the range from 4 to 25 (15.26 ± 3.97) for males and from 8 to 21 (12.93 ± 4.77) for females. Males exhibited a tendency for higher autistic traits expression than females (*t*(47) = 1.52, *p* = 0.067; two-tailed). Most important, in accord with our expectations, albeit in males only, the AQ scores negatively correlated with accuracy on the BME task (*r*(21) = −0.415, *p* = 0.024, one-tailed; effect size, *q* = 0.442) as well as on the RMET-M (*r*(21) = −0.593, *p* = 0.002, one-tailed; effect size, *q* = 0.682), whereas the negative link between the AQ scores and accuracy on the FME task only tended to reach significance (*r*(21) = −0.349, *p* = 0.052). Yet in females, correlations between the AQ scores and performance on all three tasks were non-significant (FME, *r*(24) = −0.105, *p* = 0.313, n.s.; BME, *r*(24) = 0.080, *p* = 0.355, n.s.; RMET-M, *r*(24) = 0.084, *p* = 0.347, n.s.).

## Discussion

This work was directed at the proof of concept according to which reading faces is tied with body language reading. Keeping in mind evidence for gender-specific modes in social cognition, we focused primarily on gender specificity of this link. The findings reveal that: (i) Females excel on inferring emotions from body locomotion, but not from dynamic faces. Moreover, in females only, body language reading and face reading are firmly linked. (ii) In turn, in males only, face reading is related to reading in the eyes. The outcome points to gender-specific modes in social cognition: females primarily rely upon dynamic cues in facial and bodily displays, whereas males most likely trust configural information revealed through motion.

The findings provide support for the general concept according to which efficiency of BM processing may serve a hallmark of social cognition (Pavlova, 2012). Earlier work pointed to a tie between BM processing and social cognition: individuals with aberrant BM processing also possess lower social competence, empathy, and face recognition capabilities (Sevdalis and Keller, 2011; Miller and Saygin, 2013). Our previous study (Isernia et al., 2020) was designed to untangle the ties between BM and body language reading by using strictly identical visual input and re-directing task demands either to BM processing (gender decoding based on revealing biomechanical characteristics of locomotion; Kozlowski and Cutting, 1977; Barclay et al., 1978; Cutting et al., 1978; Pollick et al., 2005) or to inferring emotions. We uncovered gender specificity of this link: males only rely upon common mechanisms supporting gender and emotion recognition through BM (Isernia et al., 2020).

### Link between reading bodies and faces

The present work helps to untangle ties between body language reading and other social cognitive abilities. For the first time, we asked whether face and body reading skills are linked. In females only, a strong association was found between body language and face reading (in terms of both accuracy and processing speed), whereas in males, processing speed (but not accuracy) of dynamic point-light faces and bodies were related to each other.

Already the developing brain is tuned to dynamic faces and bodies. As indicated by functional NIRS, in human infants aged 7–8 months, point-light faces elicit increased concentration of oxy-Hb in the right brain hemisphere (Ichikawa et al., 2010). Event-related potentials indicate that infants aged 8 months have a larger positive amplitude in the right parietal regions at latencies between 200 and 300 ms when passively viewing upright point-light BM as compared with inverted stimuli (Reid et al., 2006). Of note, a right hemispheric dominance in BM processing has been suggested already in newborn chicks (Rugani et al., 2015). Facial muscular activity alters the recognition of both facial and bodily expressions (Marmolejo-Ramos et al., 2020). Yet little is known about communication of the neural networks underpinning reading dynamic faces and bodies. As to our knowledge, the only brain imaging study investigated the relationship between reading of point-light faces and bodies in the same cohort of participants (Atkinson et al., 2012): In neurotypical adults (*N* = 17/9 females), no difference in fMRI activation elicited either by reading faces or bodies was found in the left fusiform body area (FBA) and right STS, where substantial topographical overlap occurred between face- and body-selective areas.

By contrast with women, men appear to bank primarily on structural information revealed by motion of point-light faces. Accuracy and processing speed of reading dynamic point-light faces is tightly interconnected with the reading the mind in the static eyes as measured by the RMET-M. The link in performance between these tasks is absent in females. In addition, we did not find overperformance of females on the RMET-M [in contrast to other studies conducted with the standard RMET (Baron-Cohen et al., 2001a,^2015^; Schiffer et al., 2013; Baron-Cohen, 2017; Megías-Robles et al., 2020; Kynast et al., 2021), including our own findings (Isernia et al., 2020)]. This discrepancy most likely can be explained by modifications to the standard version (see Methods section), in contrast to which the RMET-M does not heavily rely on language capabilities and is balanced in relation to the visual input. Contrary to common beliefs about female superiority on social cognition tasks (cf. Sokolov et al., 2011; Krüger et al., 2013), females did not overperform males not only on the reading through the static eyes, but also on reading point-light faces. Yet, females were better in reading point-light body language. No gender differences on a similar task were found in our previous study (Isernia et al., 2020), presumably because for the present study, the task had been modified (see Methods section).

The present study was conducted in a student sample of young adults that affords group homogeneity. Although such a population is commonly used in the field, this may represent a limitation in terms of the outcome generalizability.

### Underpinning brain networks

Brain imaging of point-light BM processing as well as detection of social interaction in Heider-and-Simmel-like animations suggests existence of gender-specific modes in brain processing of socially relevant information even in the absence of behavioral differences: gender/sex-related dimorphism may prevent behavioral differences if they are maladaptive (Pavlova et al., 2010, 2015). Likewise, differences in neural networks might contribute to the lack of gender differences in reading of the static eyes and dynamic faces in the present study. Detailed clarification of this issue calls for tailored brain imaging work.

Reading dynamic faces and bodies as well as reading in the eyes rely on the large-scale neural ensembles constituting the social brain with such topographically overlapping nodes as the face fusiform area (FFA), STS, and insula primarily in the right hemisphere (Atkinson et al., 2012; Grosbras et al., 2012; Engell and McCarthy, 2013; Dasgupta et al., 2017). For understanding a proper functioning of this network and its pathology, one has to consider changes in brain activation unfolding over time (Pavlova, 2017a). Ultra-high-field 9.4T fMRI along with a temporal analysis of blood oxygen level-dependent (BOLD) dynamics, reveals distinct large-scale ensembles playing in unison during different stages of body motion processing (Pavlova et al., 2017). Furthermore, an integrative analysis of structural and effective brain connectivity during point-light BM detection sheds light on architecture and functional principles of the neural circuitry which is organized in a parallel rather than hierarchical way: BM detection is best predicted by functional communication (effective connectivity) and presence of white-matter pathways between the right STS and fusiform gyrus (Sokolov et al., 2018). Research on the brain networks dedicated to body language reading is sparse (Heberlein et al., 2004; Atkinson et al., 2012; Jastorff et al., 2015; Mazzoni et al., 2017; He et al., 2018). By using cutting-edge analyses of effective brain connectivity, the brain networks differentiating neutral and emotional body language had been revealed: the right amygdala and midline cerebellar vermis are profoundly engaged in non-emotional as compared to emotional body language reading, and the effective connectivity between these brain structures predicts the ability to detect the absence of emotion (Sokolov et al., 2020). This outcome opens a window for studying emotional interpretation of social signals in ASD by providing the missing connection between body language reading and limbic pathways.

### Link between autistic traits and reading bodies, faces, and eyes

Arrestingly, in males solely, reading dynamic faces and bodies as well as reading in the eyes are inversely knotted with autistic traits. By contrast, these links are absent in females. (i) *Autistic traits and the RMET.* As to our knowledge, the present study is the first to report the gender specificity of a negative link between reading in the eyes and autistic traits expression. The RMET had been developed for studying some aspects of social cognition in autism (Baron-Cohen et al., 1997, 2001a,^2015^; Baron-Cohen, 2017; for recent review, see Pavlova and Sokolov, 2022b), and the most replicable and robust finding is that individuals with ASD exhibit lower RMET scoring (Del Valle Rubido et al., 2018; Peñuelas-Calvo et al., 2019; Baltazar et al., 2021). In the neurotypical population, the RMET scores are also lower in individuals with higher autistic traits expression (Gökçen et al., 2014). (ii) *Autistic traits and body language reading.* Mounting evidence points to alterations of both BM processing and affective body language reading in ASD (Hubert et al., 2007; Freitag et al., 2008; Atkinson, 2009; Klin et al., 2009; Kaiser et al., 2010; Nackaerts et al., 2012; Pavlova, 2012; Centelles et al., 2013; Mazzoni et al., 2020, 2021; Jack et al., 2021; Sotoodeh et al., 2021; for review, see Pavlova, 2012; Barton, 2021), though intact BM processing is also reported (Murphy et al., 2009). Most important, the sensitivity to BM is inversely linked both to the severity of ASD (Blake et al., 2003) and to autistic symptomatology as measured by the autistic diagnostic observation schedule (ADOS) in adolescents (Koldewyn et al., 2010). BM perception and its development may be predictable by intelligence quotient, IQ (Rutherford and Troje, 2012; Mazzoni et al., 2020). Moreover, emotion recognition in BM is reported to be not generally impaired in a sample of high-functioning (with IQ within or higher than the normal range) autistic individuals predominated by males: some emotions are recognized much better than others (Actis-Grosso et al., 2015). In ASD, some difficulties are reported also in interpreting E-Motions, i.e., affective expressions conveyed either by static faces or body postures with a high degree of perceived dynamics, forces at work (Della-Torre et al., 2021). Individuals with a high degree of autistic traits expression exhibit deficits in identifying whole-seen own body motion (Burling et al., 2019). In a sample of adults predominated by females (*N* = 57/16 males), a negative association is found between autistic traits and detection of a point-light walker’s facing (Miller and Saygin, 2013). In the same-sex twins aged 15–27 years, perception of local point-light BM (motion of single elements) rather than a global configuration is connected with heritable autistic traits (Wang et al., 2018). Yet, rather paradoxically, in preterm-born children aged 8–11 years, autistic traits are positively correlated with the ability to determine identity of walkers represented by locomotion of stick figures (Williamson et al., 2015). In neurotypical adults (*N* = 12/7 males) pooled together with autistic individuals (*N* = 12/7 males), emotion recognition through BM is reported to be knotted with scoring on the Social Responsiveness Scale (SRS, serving for detection of autistic symptoms) as well as with BM processing, and discrimination between canonical and scrambled walkers (Nackaerts et al., 2012). Here, for the first time, we report not only the negative link between emotional dynamic body language reading and autistic traits expression, but also gender specificity of this tie. (iii) *Autistic traits and face reading.* Finally, for the first time, we show the negative bond between reading of point-light dynamic faces and autistic traits expression, underscoring its gender specificity.

Notably, autism is well-known for its skewed gender/sex ratio: males are affected more often, with a ratio of about 4:1 or even greater (Hull et al., 2020; Maenner et al., 2020). Moreover, females and males are affected differently in terms of clinical picture, prevalence, and severity (Pavlova, 2012, 2017b). Female ASD is understudied, and, therefore, certain caution is needed in drawing conclusions based on male-predominant cohorts. Neurobiological mechanisms of the greater prevalence of affected males are largely unknown, though the *female protective effect* is thought to stem from a genetic predisposition for ASD, differentially impacting the female brain. Most recently, it is reported that genetic load for ASD affects functional connectivity of the salience network [the midcingulo-insular network (M-CIN), a large-scale brain network primarily composed of the anterior insula (AI) and dorsal anterior cingulate cortex (dACC) that contributes to a variety of complex functions, including social behavior, through the integration of sensory and emotional input] in boys (8–17 years old) but not in girls with and without ASD (Lawrence et al., 2020). This outcome suggests that risk genes for ASD intermingle with sex-differential processes, thereby contributing to the male bias in autism prevalence.

### Résumé

For achieving efficient social interaction during the COVID-19 pandemic, we are forced to combine social signals from different sources such as the eyes (with a face hidden behind a mask) and bodies. The present work was directed at the proof of concept according to which face reading is intimately tied with body language reading. The outcome reveals that: (i) Females excel at inferring emotions from body locomotion, but not from dynamic faces. Moreover, in females only, body reading and face reading are firmly linked; (ii) In turn, in males only, face reading is closely related to reading in the eyes as assessed by the modified version of the RMET, RMET-M. The outcome points to gender-specific modes in social cognition: females primarily rely upon dynamic cues in facial and bodily displays, whereas males most likely trust configural information revealed through motion. Arrestingly, in males solely, reading of dynamic faces, bodies, as well as reading in the static eyes are all inversely knotted with autistic traits expression. The findings are of importance for examination of face and body language reading in neuropsychiatric conditions, in particular, ASD, most of which are gender-specific. Tailored brain imaging research is required to clarify to what extent face, body language, and eyes reading share topographically and dynamically overlapping neural networks. This may be of particular value in light of the current COVID-19 pandemic. Mandatory covering faces with medical masks may lead to difficulties in social cognition and interaction (Pavlova and Sokolov, 2022a,b). As people are unable anymore to rely on the habitual information, they need to pick and pool together social signals from different sources such as eyes and bodies. In this connection, revealing bonds between reading faces, bodies and mindreading in the eyes, as well as their gender specificity is of particular value. The present work suggests that if males with autistic traits experience difficulties in reading covered with masks faces, these deficits may be unlikely compensated by reading (even dynamic) bodies and faces. By contrast, in females, reading covered faces as well as reading language of dynamic bodies and faces are not compulsorily connected to autistic traits preventing them from paying high costs for maladaptive social interaction.

## Data availability statement

The data supporting the conclusions of this article are either included in the article or will be made available by the authors upon request to any qualified researcher.

## Ethics statement

The study protocols involving human participants were reviewed and approved by the Ethics Committee at the University of Tübingen Medical School. Participants provided their written informed consent to participate in this study. Written informed consent was obtained from the individual(s) for the publication of any potentially identifiable images or data included in this article.

## Author contributions

MAP conceived and designed the study, wrote the manuscript, and supervised the whole project. SI, VR, and JK contributed to stimuli creation and programming of experiments. VR, JK, and ANS performed the experiments and collected data. MAP and ANS analyzed the data and created figures. MAP and AJF contributed reagents, materials, and analysis tools. All co-authors contributed to the writing and editing of the manuscript. All authors contributed to the article and approved the submitted version.
